# Through the Kinesthetic Lens: Observation of Social Attunement in Autism Spectrum Disorders

**DOI:** 10.3390/bs7010014

**Published:** 2017-03-18

**Authors:** Rosemarie Samaritter, Helen Payne

**Affiliations:** 1 Codarts University of the Arts, MA Arts Therapies, Kruisplein 26, 3012 CC Rotterdam, The Netherlands; 2 KenVaK Research Centre for the Arts Therapies, PO Box 550, 6400 AN Heerlen, The Netherlands; 3 School of Education, University of Hertfordshire, Hatfield AL10 9EU, UK; h.l.payne@herts.ac.uk

**Keywords:** autism spectrum disorder, social attunement, movement observation, dance movement therapy

## Abstract

This paper will present a movement-informed perspective to social attunement in Autism Spectrum Disorders (ASD). Background: Dance movement therapy (DMT) is a psychotherapeutic intervention that is used with participants with ASD in various settings. Regular clinical outcome monitoring in an outpatient setting in the Netherlands had shown positive effects on social attunement capacities in young people with ASD. However, a systematic study of the development of social attunement movement behaviors of participants with ASD throughout a DMT intervention was not yet available. Methods: A series of individual cases of DMT with young people with ASD (mean age 12.2 years.) were analyzed for changes in interpersonal movement behaviors employing video-based retrospective observation. Results: The findings were summarized in an observation scale for interpersonal movement behaviors. This scale was then tested for its applicability for the monitoring of social attunement behaviors throughout therapy. Discussion: A movement-informed perspective may be helpful to inventory changes in social attunement behaviors in participants with ASD. The relevance of a movement-informed perspective for the concept of social attunement in ASD will be discussed.

## 1. Introduction:

Autism spectrum disorder (ASD) is a diagnostic category that describes atypical developmental features with a broad range of appearances. The diagnosis ‘autism’ was introduced by Kanner in 1943 [1]. It is generally assumed that interactions of hereditary, neurobiological, developmental and environmental characteristics and structures play a role in the development of the condition [2]. The individual appearances of ASD may vary broadly, and may also depend on individual intellectual and motor abilities. Until 2013, the classification of the Diagnostic Statistical Manual (DSM) of the American Association of Psychiatrists [3] differentiated subtypes such as Asperger syndrome for high-functioning individuals, pervasive developmental disorder, childhood disintegrative disorder and Rett’s syndrome. In the DSM-5 [4] the subtypes were substituted with the global diagnosis ASD. In the diversity of appearances of ASD, three core markers have been described to be present over the spectrum. These are: (i) a diminished ability to communicate; (ii) reduced social functioning; and (iii) a preference for stereotyped routines [3,5]. In clinical practice, descriptive diagnoses are used to portray the specific personal traits of the condition for individual persons. 

Therapeutic approaches for individuals with ASD have strongly focused on the atypical development of social interactions. The development of social cognition has been considered crucial in understanding another person’s mind and in participating in meaningful interpersonal interactions such as joint attention, turn-taking or shared decision-making [6]. Interventions in social cognition in ASD aim to support the development of interactional patterns that parallel neurotypical ways of social interaction [7]. Self-advocates, by contrast, promote a perspective in which the behaviors of individuals with ASD are considered for their own specific quality of interactional intention. Following this perspective, the challenge for a neuro-typical interaction partner/therapist would be to recognize the specific interaction patterns of the partner with autism. Recent investigations into social cognition and sense-making discuss the importance of experiential knowledge that is generated through participation in social attunement processes during intersubjective interactions [8]. This enactive perspective on social cognition has also been discussed for the development of social cognition in individuals with ASD [9]. 

Social attunement is a complex phenomenon that consists of a basic attention towards an interaction partner and an intentional engagement with the other, for example during joint attention or joint actions [10]. Hence, it is the situatedness within the interaction that determines whether an action is attuned to another person or not. The movement characteristics of individuals with ASD seem to interfere with the spontaneous attunement towards interactions with another person. The timing of action responses, for example, may follow atypical patterns [11,12]. This does not necessarily imply that persons with ASD do not relate their actions to the interaction partner, but, due to the atypical character, it might be difficult for a neuro-typical person to develop a sense of relatedness from the interactional patterns that are used by the individual with ASD [13]. The role of reciprocal embodied interactions for the development of interpersonal engagement and attunement has been described by many researchers [14,15,16,17]. As intersubjective attunement seems to develop mainly through nonverbal actions, a movement perspective may be helpful to explore atypical forms of social attunement as seen in ASD [13].

## 2. Dance Movement Therapy in ASD

### 2.1. DMT: General Theoretical Background and Methods

Dance movement therapy (DMT) is a psychotherapeutic approach that uses dance and movement as experiential pathways to increase wellbeing and improve psychological or psychiatric conditions. Dance movement therapists seek to engage participants in creative movement processes and interpersonal kinesthetic experiences. The therapeutic relationship develops primarily within the kinesthetic attunement between patient and therapist. As in dance as a cultural practice, the social engagement in the therapeutic alliance develops through shared dance actions [18]. The moment of dancing generates an immediate sense of the experiential quality of shared movement themes [19]. The potential to express and share inner states through movement with observers or other dancers defines dance as a specific, bodily-experienced aesthetic practice [20] which has an underlying assumption that ‘...a potential cannot be given or rehearsed—a potential has to be...found...’ [21]. Dance-based methods in DMT may cover a broad range, from structured movement activities to improvised movement processes, but all these methods share the basic assumption that dance-based activities support and develop an integrated bodymind engagement and interpersonal relating [22].

### 2.2. DMT with Participants with ASD

DMT approaches with participants with ASD have been mainly described through case studies. In recent years, the practice-based literature is complemented by research studies on the effectiveness of DMT interventions in ASD [23,24,25].

Despite the diversity in settings, DMT interventions for participants with ASD are characterized by some common features. All approaches share the premise that participants should be met in their personal movement patterns and possibilities to interact. DMT stresses empowerment of the participants’ capacities to move and seeks to develop already present potentials towards a broader range of movement possibilities. The therapist will specifically support nonverbal interaction. Although atypical sensory-motor features may arise during DMT with participants with ASD, these will generally not be taken as disturbances by the therapist, but instead as movement material that can be explored for their interactional potential [26].

As the basic intention of the therapist is to meet the participants with ASD in their personal movement impulses, the therapist will carefully investigate occurring movement actions. It is common practice in DMT to analyze participants’ actions with a specific movement observation system. The most frequently used observation systems are Laban Movement Analysis [27] and the Kestenberg Movement Profile [28]. 

With ‘mirroring’ interventions, the therapist seeks to get as close as possible to the participant’s movement patterns [29]. Mirroring in this case is a flexible and playful reflection by the therapist of the participant’s movements or movement themes. Case studies conducted with mainly mirroring interventions with children with ASD report effects on the sensory-motor regulation of the child, as well as the expressive relating with the environment or a movement partner [30]. These outcomes have been confirmed by research studies with children [31] and young adults [32].

The DMT method used in the clinical samples that were used for this study has been described as the Shared Movement Approach (SMA) [22]. In this approach the therapist works with a single participant in dyadic DMT sessions. The movement interactions between therapist and participant are taken as improvisational dance patterns [33,34]. The movement actions of the participant are reflected by the therapist through an immediate responsivity that takes the participants’ momentum towards shared movement activities [26]. All movement actions of the participant are answered from the perspective of their interactional potential, with the intention to take them towards dialogical movement interactions. The therapist invites the participant into an active co-creation of the shared movement themes. The therapeutic dyad develops throughout the improvised dance as a living dynamic system that is mutually regulated by both movers through the movement contributions they add to the shared dance.

## 3. Movement Observation and DMT/ASD

### 3.1. Movement Observation and DMT

The somatic experiential encounter between therapist and participant in DMT entails specific dynamics, which can be difficult to measure and quantify. Laban Movement Analysis (LMA) is an observation-based system that categorizes movements under the aspects of use of *body*, *space*, *effort* and *shape* [35]. Within these observation categories the specific qualities of the movement patterns are described as combinations of spatial, time, weight and energy aspects. The strength of this system is the non-interpretative registration of movement qualities, regardless of any developmental or psychological features. This allows for the therapist to monitor the participant’s (re)occurring movement patterns, which may serve as a point of reference for the movement interventions and for the evaluation of changes in the personal movement profile during therapy. 

### 3.2. Movement Observation and ASD

Individuals with autism may show atypical features of sensory-motor functionalities throughout development [36,37,38,39]. In the clinical treatment of children and adolescents, the sensory-motor maturation is examined through functional analysis with developmental test materials like Southern California Sensory Integration Test [40], Marburger Körperkoordinationstest, Entwicklungsraster Psychomotorik [41] and the PsyMot [42].

The dance movement therapist will add a focus on the specific, individual movement repertoire of a participant’s functional analysis, regardless of whether the movement development shows typical or atypical traits. Movement analysis enables dance therapists to observe, describe and notate movement qualities systematically and compose a movement profile of the client. 

Case studies have described some similar movement specifics for young participants with ASD [43,44,45,46]. Sossin and Loman [47], both working with the KMP, described participants with ASD showing a tendency to use a neutral flow of body shape, which gives the impression of a lack of animation. They frequently observed a preference to move with highly isolated tension, often resulting in a lack of continuity of movement and apparently unrelated adjustments of movements or clashes during movement adjustments. Partial stabilization of body parts and shaping activities, like organizing their posture around that of a partner, seemed to be less present in participants with ASD [48]. 

The ‘Behavior Rating Instrument for Autistic and other Atypical Children’ (BRIAC) developed by Kalish and colleagues [49] evaluates among other domains the child’s movement development. 

These Laban-based methods have in common that they document how the movement is performed and then describe the movement quality, but they do not necessarily capture the social components of the movement actions, nor do they provide a quantitative structure to monitor changes throughout therapy or for outcome evaluation. 

With the growing need for evidence-based practice, movement-specific evaluation tools would be needed to cover the domain-specific content of dance therapy activities. In the past, only a few studies used movement behaviors for outcome evaluation of social interaction in participants with ASD [50,51].

The purpose of this study was to monitor changes in social attunement of young participants with ASD during DMT and to identify specific, observable movement markers that were present during these changes. The obtained interactional movement markers were expected to be useful to the inventory of nonverbal aspects of the participants’ capacities for social attunement. Observation with a structured set of interactional movement markers was also expected to support the systematic evaluation of effects of DMT on social attunement in young participants with ASD. 

## 4. Methodology

The study was conducted with a mixed methodology. A retrospective analysis was applied to video-materials of dyadic DMT with young participants with ASD. Retrospective observation facilitates the exploration of experiential phenomena as they occur in behavioral changes over time [52] and may support the construction of significant events for a domain of interest [53]. The observation of the interaction between participant and therapist was expected to allow the identification of the specific interactional movement behaviors used by the participants. The domains of interest were the participants’ nonverbal intersubjective engagement and their attunement behaviors within the therapeutic dyad.

The collection and analysis of observational data was performed with a Grounded Theory Approach (GTA) [54]. This qualitative research procedure follows a systematic sequence of analysis—for example open, axial and selective coding [55]—to reveal implicit structures of verbal or experiential data. GTA was applied to collect interactional movement behaviors and analyze them for core themes and structures. Because the researcher had also been the therapist at the time, the observational perspective was informed by her experiences in the clinical setting. This choice was based on the understanding of the investigation as a heuristic research process, during which the effort to understand the underlying structures of a lived situation (in this case of the clinical DMT) is understood as a complex, experientially informed way of thinking and sense-making [56].

Several steps were taken to account for the quality of content validity of the obtained observational categories and to avoid biased interpretation during the GTA procedures (Section 4.5).

### 4.1. Procedures

The video materials used in this study were recordings that had been produced for the evaluation of the therapeutic outcomes of clinical DMT sessions with young participants with ASD in an outpatient hospital setting. All cases had been evaluated positively after termination of the therapy by parents, participants and caregivers for the development of social attunement in the participant. Therefore, it was expected that meaningful changes in social attunement would also be observable during the recorded DMT sessions. 

Video vignettes were prepared for observation by anonymizing the samples and providing password-protected usage. The annotation procedures were partly performed by hand with paper and pencil, but mainly with the free software package ELAN [56], that initially has been developed for the linguistics department of the Max Planck Institute, Nijmegen (NL) [57].

The materials were first scanned with an open-coding procedure to detect and tag scenes that contained changes in the interaction from the participant towards the therapist. These scenes were analyzed for the movement components that constituted these changes. GTA was used to analyze the movement markers for similar structures and themes. 

The obtained movement markers were then critically annotated by external movement analysts and tested for inter-rater agreement on the contents of the categories. Inter-rater agreement also served as a measure to decide upon the suitability of two different coding procedures applied with the obtained movement markers. 

In a last step, the movement markers were used in a selective coding procedure to analyze the changes in interactional movement profiles over four time points (TP) throughout therapy in four single cases of dyadic DMT. 

### 4.2. Participants

From the available video material four cases were selected for their similarity in duration of therapy and available video vignettes throughout therapy process. All participants had been diagnosed with ASD by a child psychiatrist and had been referred to DMT by a multidisciplinary treatment team or school psychological service. Participants were two girls and two boys, with a mean age of 12.2 years (SD 3.8). All participants attended a school for children with special needs. DMT was offered in the outpatient unit of the child and youth psychiatric department of a Dutch National Health Service hospital. DMT was the only therapeutic intervention the participants followed at the time. 

Participants and parents had given their informed consent for the use of the video materials for research purposes after termination of the therapy. Ethical approval for the project was sought through the appropriate institutional procedures. All therapies were conducted as dyadic DMT with the researcher being the therapist in all cases. 

Participants in the inter-rater procedures were six trained movement analysts, including the researcher, with one a psychologist who had extensive training in movement analysis. Analysts had been recruited through professional DMT networks in the Netherlands. All six analysts participated in an expert circle and the testing of appropriate annotation procedures. Two analysts participated in the observation of selected video vignettes for the evaluation of interpersonal movement behaviors throughout therapy in four single cases of DMT with participants with ASD. 

### 4.3. Data-Collection

Firstly, an open-coding procedure [54] was used to select scenes that contained changes in the interaction between participant and therapist. Verbal actions were not considered during these observations. The collected moments of change were taken as raw data. These data were analyzed (employing LMA) for the movement components present in the actions of the participants. In a second step, which covered an axial coding procedure [54] these actions were coded for similar structures and overarching aspects. 

The obtained structural categories were used to collect data on inter-rater agreement regarding the contents of the movement categories and annotation procedures. The set of movement items was piloted for the observation of interpersonal movement behavior in a small clinical study on different types of DMT interventions [58].

The edited movement categories were then used for a selective coding procedure on vignettes from four single cases at four time points (TPs) during the therapy process. Samples from the original material were selected with a randomization procedure and did not contain any information about the progression of the therapy. The annotations of two raters were analyzed for inter-rater agreement. The observational data were then analyzed for the movement profiles for the individual participants for each TP.

### 4.4. Data-Analysis

In the collected scenes, the occurring movement actions were analyzed for their properties with LMA based observation. After the first try-outs and the first expert circle of movement analysts, the categories were fine-tuned in view of their applicability during video-based movement observation. 

Movement actions were grouped into categories of movement direction, facial orientation, body/body part direction, weight engagement individually, weight engagement with a partner, weight regulation with a partner, synchronization in rhythm and synchronization in phrasing.

The eight movement items were grouped under three overarching themes: ‘spatial orientation’, ‘weight engagement’ and ‘synchronization in time’ (Table 1). The movement markers were defined to indicate *spontaneous movement behaviors initiated by the participant*, meaning that behaviors should be intentionally directed from the participant towards the therapist in contrast to behaviors that would have been predefined as social behaviors and then trained through instruction.

The single categories could cover quite a variety of appearances of a movement action. The item *movement direction*, for example, was found applicable to mark a movement of a hand directed towards the interaction partner (the therapist) as well as a full body movement directed towards them. The perspective of the social attunement of the participant with ASD was taken as the guiding conceptual principle. The fact that a movement was directed towards (or away from) the therapist was considered to be more important than the differentiation on the body level in partial or full body involvement. 

The item *weight engagement* was discussed among the movement analysts for the use of passive and active weight. An example of the use of passive weight could be observed when the participant was leaning on the therapist. An example of the use of active weight could be observed when the participant engaged strong weight while moving towards the therapist. The final definition of the category covered active as well as passive weight. This choice was made in view of the conceptual perspective of social attunement. The fact that the participant was engaging weight in relation to the therapist was considered more relevant in view of social attunement than the differentiation into separate categories for active or passive use of weight. 

The final scale was tested for two different annotation procedures with six movement analysts in a separate study [20]. An interval procedure [59] yielded a satisfactory inter-rater agreement [60], which was calculated with Cohen’s kappa [61] at *k* = 0.752.

The movement markers were then used in a selective observation procedure with two movement analysts on video vignettes that were selected from the available materials in the four individual cases of DMT. For each case, four vignettes from similarly distributed TPs over the therapy process were selected from the available video materials. To account for the random selection of video vignettes, the middle five minutes of the video material from a recorded session were selected for observation. For this procedure, only moments when both participant and therapist were visible in the scene were considered. The annotations were performed with an interval procedure. Analysis indicated for each of the five minutes whether a movement category was present. This yielded quantitative data on the number of minutes in which the single-movement behaviors were present. From these data, profiles of social attunement behaviors were composed for each TP. The profiles of each TP allowed for within-subject comparison between TPs as well as between-subject comparison at the different TPs. A non-parametric procedure was applied for a preliminary exploration of statistical trends for the development of movement behaviors over the four TPs. 

### 4.5. Quality Issues

During the selection procedures, journal notes were kept by the therapist-researcher to conceptualize and model the experiential data in the perspective of social attunement in dance and movement and in social attunement in ASD. GTA reflective practice of *memoing* [54] was used to annotate the observational data critically. To apply a non-biased reflection on the obtained movement categories they were piloted by observers other than the researcher and to other materials than the vignettes used in this study. 

The obtained movement items were related to the research literature on social attunement and interaction in ASD. Studies on the effect of imitation on nonverbal social behavior in participants with ASD were found to mention body-related observation items, for example *eye gaze, touch* and *proximity to another person* [62,63,64,65,66,67], but did not cover a comparable spectrum of movement *actions* as the markers found in this study.

In the development of new observational categories, validity issues are of major concern [68]. In this study, inter-rater agreement calculation served to test for agreement between raters on the content validity of the movement categories and the construct validity of the annotation procedures [69]. During the final selective-coding procedure, the therapy vignettes were presented in a random order, to blind the raters for the information on the therapy progress.

## 5. Results

### 5.1. GTA Procedure

The results of the GTA procedures were summarized in a movement observation scale. The term Social Engagement and Attunement Movement (SEAM) was introduced for the interpersonal movement markers. The specification of the categories and annotation procedures were described in a manual. 

### 5.2. Selective Observation

The selective observation with the obtained markers showed in all four cases an overall increase of SEAM behaviors over the four TPs. The changes occurred within single categories as well as over the number of SEAM behaviors involved during the observed movement actions. 

All participants showed an increase of complexity of SEAM behaviors towards the end of the therapy, with all profiles showing a combination of spatial, weight and time movement aspects at TP 4, which was not the case in the profiles at TP 1. Within-category analysis showed an increase for all eight markers over the four TPs. The development of SEAM behaviors over the four TPs followed a similar pattern structure for all cases.

**Figure A1:**
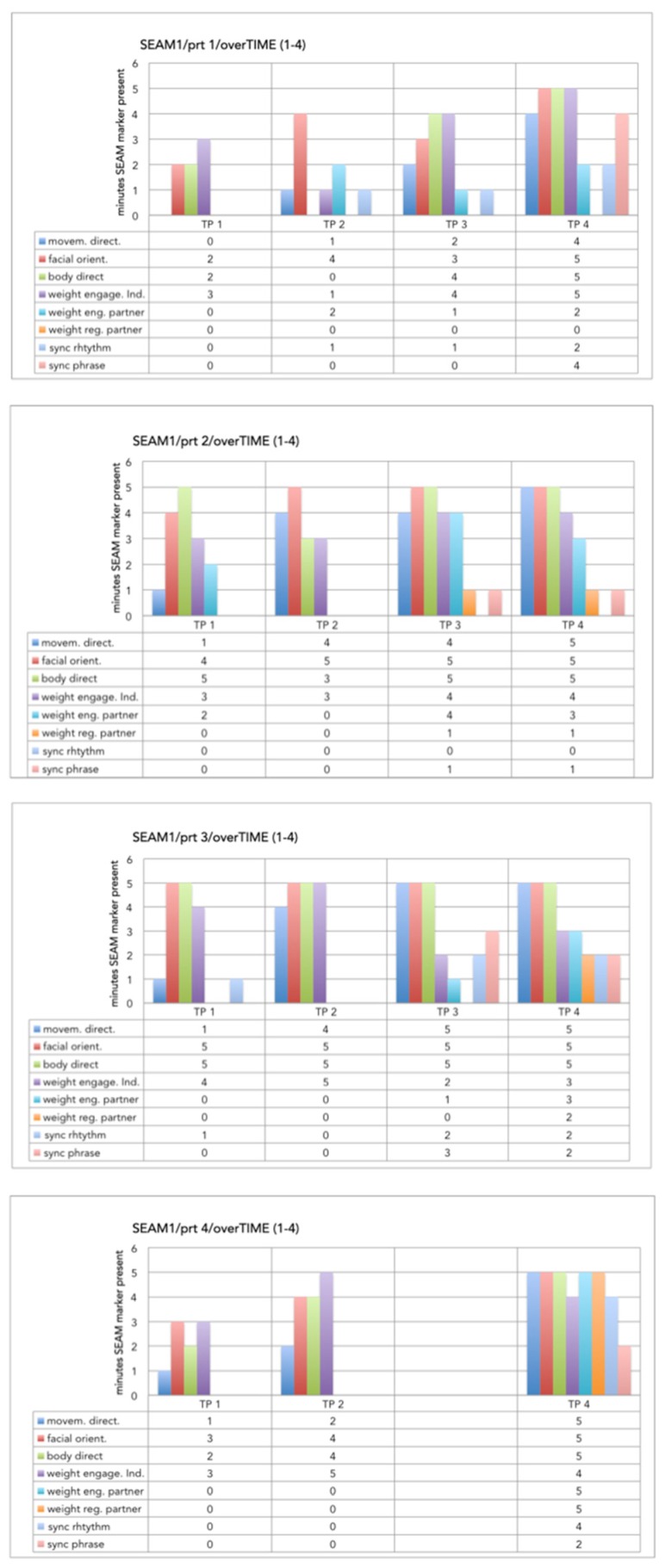
To Show a graphical summary of the individual profiles of SEAM scores at TP1, TP2, TP3, TP4.

**Figure A2:**
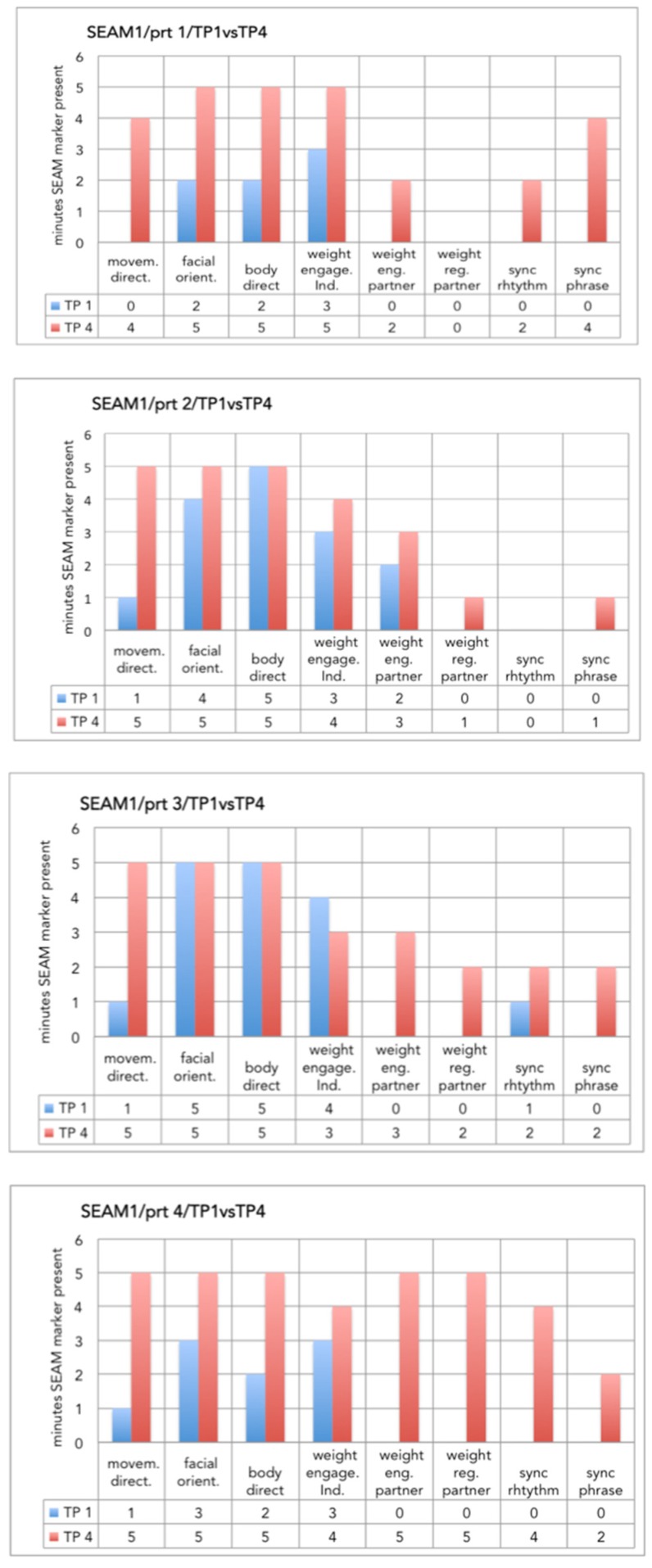
To show a graphical summary for all participants of the changes in scores for single SEAM categories between TP1 and TP4.

The behavior ‘spatial orienting’ was already present at TP 1 and throughout all other TPs for all participants. Behaviors related to the use of weight were less present at TP 1. None of the profiles showed ‘weight regulation towards a partner’ at TP 1. ‘Weight engagement towards a partner’ was only present in one profile at TP1. The behavior ‘synchronization in phrasing’ was not present in any of the profiles at TP 1, ‘synchronization in rhythm’ was only found in one profile at TP1. These behaviors developed (increased) throughout therapy process in all cases, with two profiles showing all SEAM behaviors at TP 4 and two profiles showing seven of the eight SEAM behaviors present at TP 4 (graphical profiles (Figure A1 and Figure A2) in Appendix A).

**Figure 1:**
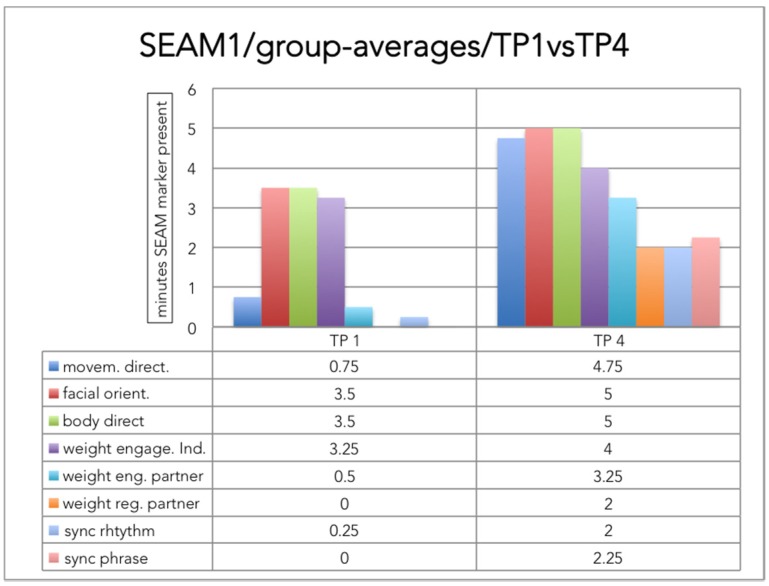
To show a graphical summary of differences in the average group SEAM profile between the start and end of dance movement therapy (DMT). The SEAM categories are presented in columns; the height of the columns indicates the average number of minutes that a SEAM behavior was present during the observed five minutes’ samples.

Over the four TPs, all profiles showed a trend for the orienting behaviors to occur prior to behaviors of weight engagement and regulation towards the therapist. The synchronizing behaviors occurred only after weight engagement and weight regulation had appeared in the participant’s interpersonal movement behavior. This structure was particularly visible in the comparison of the group averages of TP 1 and TP 4, as shown in Figure 1.

Due to the small number of cases involved in this study, the statistical analysis was limited to a preliminary analysis with non-parametric measures. A Friedman test was applied to the group averages of the four TPs. With a Chi-square value *χ*^2^ = 7.95, it yielded with a significance level at 0.05 a *p*-value of *p* = 0.0028. A Wilcoxon Signed-Rank test indicated with Z = −2.527 and with a significance level at 0.05 a *p*-value of *p* = 0.012 a significant increase for the group averages of SEAM behaviors between TP 1 and TP 4.

## 6. Discussion

From a movement perspective, interpersonal attunement may be understood as the interplay of several directly observable behavioral components. The interactional intention may be demonstrated in the active orienting toward the other, together with active engagement and regulation of impulses towards the other. The interplay of these aspects as observed in the SEAM behaviors during DMT conveys a movement-informed perspective on social attunement in participants with ASD.

The successive emergence of spatial orienting, weight engagement, weight regulation and synchronization in timing may be considered developmental steps that, taken together, contribute to a high complexity of interpersonal engagement and attunement during shared movement actions. This emerging complexity may contribute to a (new) experiential quality of interpersonal actions. 

In view of enactive social learning, these processes may add to the experiential means of the participant with ASD to responsively move with a partner. The co-regulation of the shared movement situation is experienced through kinesthetic perceptions. The embodied quality of these experiences may facilitate transfer to interactional contexts other than therapy, because nonverbal interactions with peers or caregivers are characterized by similar dynamic structures [70]. Reports of teachers on changes in attunement behaviors of the participants towards peers during open play situations also point in this direction.

For the dance therapist, the SEAM markers may help to observe and analyze the development of interpersonal attunement behaviors of the participant closely throughout the therapy process and to tailor the interventions during shared movement improvisations accurately.

In the starting phase of the therapy, participants’ SEAM behaviors showed lower complexity in the sense that not all movement categories were present during the interactions. The initial presence of spatial orienting in all cases seemed to fit with the fact that all participants were at a functional level that allowed them to attend therapy in an outpatient setting. The integrated use of social orienting, engaging and synchronizing qualities during the final phase of the therapy was understood as fully attuned interpersonal movement behavior. The developmental pathway of SEAM behaviors throughout therapy could also be related to the development of interactional patterns in the early dyad between young children and caregivers [71]. 

With the increased complexity in the SEAM patterns, the participants seemed to gain more agency and regulation in the movement interaction with the therapist. The higher complexity of SEAM behaviors also delivered more cues for interpersonal responsive movement interventions for the therapist. These results match with the therapy outcome evaluations of parents and other caregivers who reported their children to be better attuned during interactions with peers and adults. 

### Limitations

The satisfactory agreement between movement analysts points towards content validity of the SEAM categories. However, the construct validity of the SEAM markers as an observational scale for social attunement behaviors would need further testing against standardized instruments [68]. Also, the robustness of the scale as a quantitative research tool would need further validation [72].

The small number of cases involved in this study demands a prudent interpretation of the results. Further studies with a larger cohort would be needed to confirm these findings.

All sessions observed for this study had been conducted by one therapist. Replication between therapists and over more cases could strengthen the results. Further research might also show if observations with the SEAM markers during DMT by the therapist would have an impact on the effectiveness of the DMT intervention.

## 7. Conclusions

Retrospective analysis of movement interactions between participants with ASD and the therapist during DMT revealed movement behaviors for the theoretical construct of social attunement [73,74,75]. Movement observation with the SEAM scale seemed to reveal developmental potentials of social attunement rather than pathological shortcomings in participants with ASD. The observation of SEAM behaviors may contribute to therapists’ understanding of the developmental pathways of social attunement in participants with ASD as they occur during DMT. In this study, the development of interpersonal movement behaviors showed a specific sequence from orienting, via engagement towards synchronization. Further research may investigate if this developmental sequence of attunement behaviors is characteristic in participants with ASD. A movement-based observation tool may be helpful to capture an experiential perspective to interpersonal relating and to indicate potential cues for the development of social attunement between individuals with ASD and typical interaction partners. From the current study, we may conclude that the SEAM observation scale seems suited to monitor changes in nonverbal interpersonal relating behaviors during DMT. The scale may be helpful to monitor a participant’s development during the therapy process as well as for therapy evaluation.

## Figures and Tables

**Table 1:** Overview of interpersonal movement behaviors.

Overarching structures	Social Engagement and Attunement Movement (SEAM) behavior as observed:
Observed interpersonal movement behavior in terms of *space*	➢ Participant orients face towards partner ➢ Participant direct body/parts towards partner ➢ Participant directs movement towards partner
Observed interpersonal movement behavior in terms of *weight*	➢ Participant engages weight during individual movement ➢ Participant engages weight towards the partner ➢ Participant regulates weight towards partner
Observed interpersonal movement behavior in terms of *time*	➢ Participant synchronizes movement rhythm with that of partner ➢ Participant synchronizes movement phrase with that of partner
